# Explicit Simulation of Circular CFST Stub Columns with External Steel Confinement under Axial Compression

**DOI:** 10.3390/ma13010023

**Published:** 2019-12-19

**Authors:** Faesal Alatshan, Siti Aminah Osman, Fidelis Mashiri, Roszilah Hamid

**Affiliations:** 1 Smart and Sustainable Township Research Centre, Faculty of Engineering and Built Environment, Universiti Kebangsaan Malaysia, Bangi 43600, Selangor, Malaysia; p94378@siswa.ukm.edu.my (F.A.); roszilah@ukm.edu.my (R.H.); 2 Civil Engineering Department, College of Engineering Technology, Houn 61160, Libya; 3 School of Computing, Engineering and Mathematics, Western Sydney University, Sydney, NSW 2751, Australia; F.Mashiri@westernsydney.edu.au

**Keywords:** concrete filled steel tube (CFST), ring/spiral confinements, explicit simulation, stub columns, finite element analysis (FEA), axial compression

## Abstract

Concrete-filled steel tube (CFST) structural members have been widely used in engineering projects for their superior strength and ductility. However, the different lateral dilation characteristics between concrete infill and steel tube have caused imperfect composite interaction during the early loading stage. To overcome this issue, external steel confinements in the form of rings and spiral were previously suggested to minimise the lateral expansion of the steel tube and enhance the concrete confinement effects. This study presented the analytical behaviour of circular CFST short columns with an external ring or spiral confinements which are subjected to axial loading. An explicit finite element (FE) model was developed and verified based on previous experimental findings. Besides that, this study analysed the failure modes, axial load–strain relationship, stress distributions, and bond strength of the composite column components. Parametric analysis was also undertaken to evaluate the impact of material strengths, total steel ratio, and diameter-to-thickness ratio. The results suggest that the use of external steel confinement can enhance the compressive behaviour of CFSTs better than increasing the thickness of the steel tube when using the same steel ratio. Finally, simplified design formulations were developed to accurately calculate the ultimate capacity of CFST columns with and without external steel confinement.

## 1. Introduction

Over the past few decades, there has been an accelerating increase in employing concrete-filled steel tubes (CFST) in various types of engineering structures, including industrial workshops, bridge piers, power transmitting poles, and high-rise buildings [1,2]. It is well known that CFST technology has various advantages compared to conventional technologies, including empty hollow structural section (HSS) or reinforced concrete (RC). The concrete infill improves the mechanical strength of the member by preventing or delaying steel tube inward buckling. Moreover, the concrete confinement by steel tube can increase the member ultimate strength, ductility, and seismic behaviour [1]. CFST structural elements have smaller section sizes and do not require any formwork, that can provide more sustainable elements with lower construction costs. Additionally, CFST members offer better fire resistance compared to empty HSS [3].

However, the perfect performance of CFST columns requires strong concrete-steel interaction bond to guarantee that they work together as one composite element. The imperfect interaction in CFSTs can significantly reduce their strength [4,5]. Consequently, the use of expansive concrete or adding internal or external restraint are the two solutions that have been previously suggested to improve the bond carrying capacity of CFST elements. Adding expansive additives to concrete admixture can reduce the separation between concrete and steel tubes, which result in improving ultimate strength, creep behaviour, and bond stress [6,7]. However, this approach can only solve the influence of concrete shrinkage and temperature changes, while the impact of differential dilatation between steel tube and the concrete infill at early loading stage will occur even when using expansive concrete.

Because of the difference in Poisson’s ratios between concrete (*ν* = 0.18) and steel (*ν* = 0.3), bond delamination failure may occur between the interaction surfaces of the two materials at the elastic stage. This failure will weaken the impact of steel confinement of the infilled concrete. In other words, as the concrete Poisson’s ratio is less than the steel, the lateral expansion of infilled concrete will be smaller than the steel tube at the elastic stage. Hence, no composite interaction between the two components will occur before the spreading of micro-cracks in the concrete and the beginning of inelastic outward buckling of steel [8,9]. Therefore, a different approach was suggested to strengthen the bond behaviour of CFST by utilising steel stiffeners.

Internal stiffeners including plate ribs [10,11,12], tie bars [13,14,15,16,17], and curling ribs [18,19] have been utilised to improve the composite interaction of CFST columns. It was found that internal stiffeners could improve the CFST columns strength with better deformation characteristics. Ductility can be also improved using appropriate types of stiffeners. Tao et al. [5] proposed another type of internal stiffeners using welding ring on the inner surface of the steel tube. The results illustrated the effectiveness of using these stiffeners in CFST compared to welding shear connectors and utilising expansive concrete.

Practically, the installation of internal stiffeners in small diameter tubes is complicated, especially welding stiffeners on the curved surfaces of circular columns. Moreover, the setting up of tie bars necessitates drilling holes in the tube that can result in generating high stress concentration at the locations of tie bars. In addition, internal stiffeners may inhibit the flow of concrete inside the tube, which will increase the cost due to the need for using high-performance concrete.

Consequently, another mechanism of external confining scheme was suggested to overcome the limitations of CFST columns with internal stiffeners. CFST external confinement including fibre reinforced polymers (FRP) strips [20], steel rings [21], and steel spirals [22] were previously introduced to decrease the lateral dilation of CFST columns. Additionally, external confinement can provide additional confinement of the structural elements to enhance its resistance and ductility. In contrast, structural members confined by FRP had shown sudden brittle failure because of the linear elastic performance of FRP wraps [23,24]. In addition, it is a costly material and has poor fire resistance.

Despite the considerable research efforts that have been previously performed to understand the behaviour of CFST columns, there is still a lack of studies that evaluated the performance of CFSTs with external steel confinements. Lai and Ho [21] attempted to enhance the mechanical performance of 62 CFST columns with external steel rings confinements which were subjected to axial loading. Their results revealed that the ring-confinement could control the lateral deformation of CFST columns and thus increase the ultimate capacity of CFSTs until up to 48.6%. In another study, Lai and Ho [22] investigated the axial performance of 24 CFST columns confined using external steel spirals. They found that the load-carrying capacity could be improved by up to 36.5% compared to unconfined CFST columns. However, there was no study that evaluated the efficiency of external confinements of CFST columns compared to increasing the thickness of the steel tube, or even comparing the two types of external confinements (rings and spiral). Additionally, no finite element analysis (FEA) was undertaken to investigate the behaviour of CFST elements with external confinements. This study proposes a verified finite element model for CFST short columns with ring and spiral external confinements. This model was employed to perform a parametric analysis and highlight the significance of different design parameters.

## 2. Finite Element Analysis (FEA)

FEA using ABAQUS software [25] was developed to explore the characteristics of CFST short columns with external ring and spiral confinements. Four main components were considered to establish the proposed model namely the involving concrete, steel tube, external confinements, and the interaction between the elements.

### 2.1. Mesh and Element Type

This study used 8-node linear solid element (C3D8R in ABAQUS) with reduced integration and three degrees of freedom at each node to simulate the infilled concrete. In the literature, the steel tubes of CFST structural elements were usually simulated by four-node doubly curved shell element (S4R in ABAQUS) to capture the compressive deformation and local buckling [26]. However, it was found that the use of either shell or solid elements can produce results that capture local buckling and deformation successfully. In addition, utilising shell elements can make the model more sensitive to excessive distortion. Additionally, as there was no impact in using shell elements to decrease FEA computational time (CPU time), the C3D8R solid elements were utilised to simulate the steel tube. For the external rings and spiral steel confinements, this study used two-node beam elements with linear interpolation (B31 in ABAQUS).

**Figure 1:**
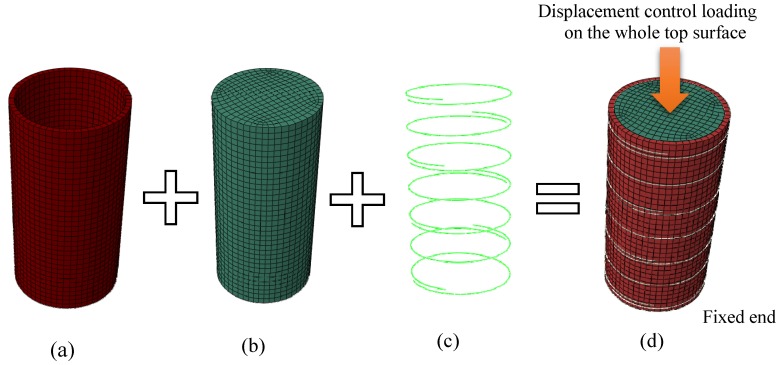
Typical mesh generation model: (**a**) steel tube element, (**b**) core concrete element, (**c**) external confinement, and (**d**) FE model.

Mesh convergence investigations were carried out to determine the suitable mesh density to provide precise results within reasonable computation times. Element mesh size was taken as D/18 for the whole element, where D is the outer diameter of the specimen. Figure 1 shows the detailed model mesh of a typical specimen.

### 2.2. Boundary and Loading Conditions

A vertical compression axial loading was simultaneously applied to the top surface of concrete and steel through the displacement control option available in the ABAQUS library. The top and the bottom end of the specimens were restrained against all degrees of freedom, excluding the vertical displacement of loaded top end. Figure 1d illustrates the loading and boundary conditions of the FE model.

### 2.3. Interactions

The contact between the inner surface of the steel tube and concrete was defined using the ‘surface-to-surface contact’ element available in ABAQUS library. Different friction coefficients (*μ*) were investigated and it was found that the value of *μ* did not have a significant impact on the results, which was expected for simultaneously loaded specimens. However, the value of *μ* = 0.6 was taken as recommended by Tao et al. [27]. The inner steel surface was assigned as master surfaces and the concrete surface was assigned as the slave. The ‘tie constraint’ was employed to specify the welding interaction between the outer surface of the tube and the external confinements.

### 2.4. Step Type

**Figure 2:**
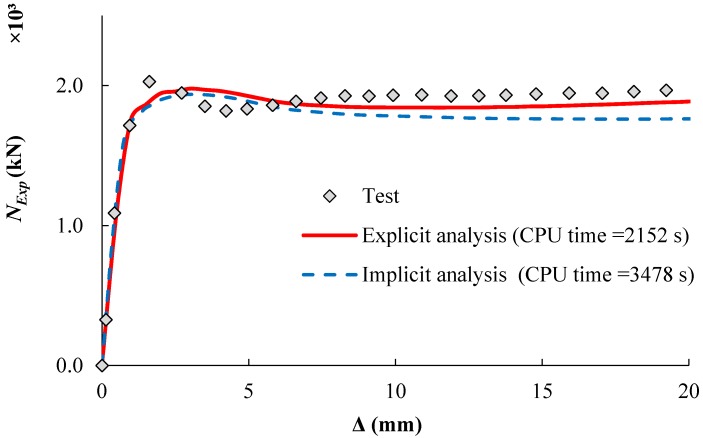
Load-displacement curves with different step types.

The implicit solution using ‘static general step’ available in the ABAQUS library has been widely utilized in CFST simulations. However, this study adopted the ‘dynamic explicit step’ to minimise computing time (CPU time) of the analysis. The use of ‘static general step’ is computationally expensive where a large number of iterations are required to compute the solution. Conversely, explicit analysis is less complicated in dealing with models with complex contact and material properties. Figure 2 shows a comparison between the implicit and explicit analysis approaches. The implicit analysis requires an inversion of stiffness matrix at each increment to meet the equilibrium conditions of the internal resistance forces with the externally applied loads. This approach results in a large number of iterations, which are too computationally expensive for complicated models. The explicit approach does not check the equilibrium at the end of each increment of time and it calculates the solution from the kinematic state of the previous increment.

In explicit analysis, choosing the appropriate values of loading rate and mass scaling can significantly shorten the simulation time. Table 1 shows the impact of using different loading rates and mass scaling factors in modelling the specimen (CR12.5-5-114-120 [21]). Any increase in the mass scaling or loading rate can reduce the required number of modelling increments, which will speed up the computational time of the simulation. On the other hand, excessive mass scaling or loading rate may affect the accuracy of simulation results.

**Figure 3:**
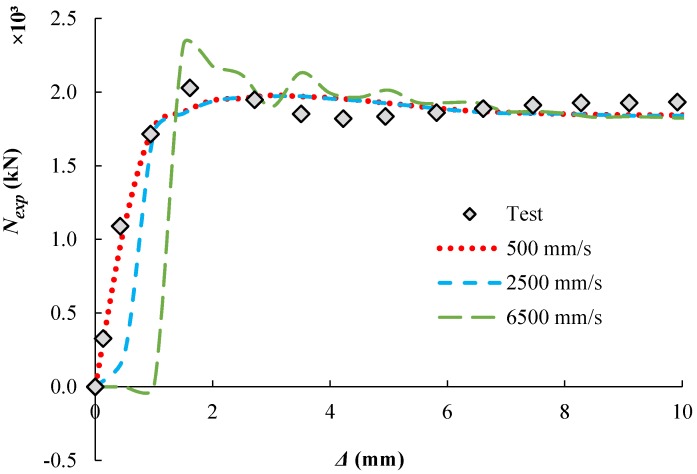
Effect of loading rates (CR12.5-5-114-120 mass = 10).

A mass scaling factor of 10 was applied to the whole model as suggested by Hassanein et al. [28] while different values of loading rates were examined. Figure 3 shows the predicted load-displacement relationship of the specimen (CR12.5-5-114-120 [21]). This study adopted the loading rate of 500 mm/s which results in good agreement with the experimental results.

### 2.5. Material Model of the Steel Tube

**Figure 4:**
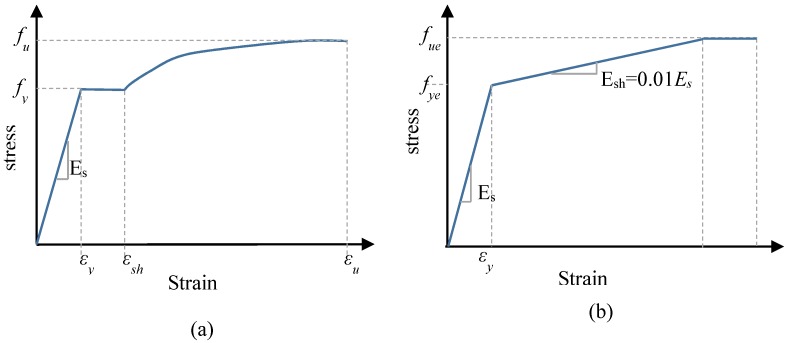
Stress–strain relations of steel. (**a**) Steel tube, (**b**) External steel confinements.

Figure 4a shows the bilinear plus nonlinear hardening material model which was proposed by Yun and Gardner [29] and adopted in this simulation to represent the steel tube stress–strain relationship. This model was established based on a large set of experimental stress–strain data to provide more accurate prediction than other models. The model requires three main parameters to describe the full-range of stress–strain relationship. Firstly, the elastic modulus of steel (*E_s_* = 200 GPa) was taken as recommended by Tao et al. [27]. Secondly, the steel tube yield capacity (*f_y_*) was adopted as reported in the experimental data [21,22,30]. The third parameter is the steel ultimate strength (*f_u_*) which was calculated according to the following Equation (1) suggested by Tao et al. [31].

(1) $$f_{u}=\{\begin{array}{cc} \left[1.6-2\times 10^{-3}\left(f_{y}-200\right)\right]f_{y} & 200MPa\leq f_{y}\leq 400MPa\\ \left[1.2-3.75\times 10^{-4}\left(f_{y}-400\right)\right]f_{y} & 400MPa\leq f_{y}\leq 800MPa \end{array}$$

Yun and Gardner’s model [29] that was adopted in this study, can be summarised in the following series of equations:

(2) $$\sigma (\varepsilon )=\{\begin{array}{lll} E_{s} & \mathrm{for} & \varepsilon <\varepsilon_{y}\\ f_{y} & \mathrm{for} & \varepsilon_{y}<\varepsilon <\varepsilon_{sh}\\ f_{y}+\left(f_{u}-f_{y}\right)\left[\frac{0.4\;C+2\;C}{{\left(1+400\;C^{5}\right)}^{\frac{1}{5}}}\right] & \mathrm{for} & \varepsilon_{sh}<\varepsilon <\varepsilon_{u} \end{array}$$

where:

◾ $C=\left(\frac{\varepsilon -\varepsilon_{sh}}{\varepsilon_{u}-\varepsilon_{sh}}\right)$
◾ $\varepsilon_{u}=0.6\left(1-\frac{f_{y}}{f_{u}}\right)\;\mathrm{but}\;\varepsilon_{u}\geq 0.06$
◾ $\varepsilon_{sh}=0.1\frac{f_{y}}{f_{u}}-0.055\;\mathrm{but}\;0.015\leq \varepsilon_{sh}\geq 0.03$

$\varepsilon_{u}\;\mathrm{and}\;\varepsilon_{sh}\;$ are the ultimate strain and the strain-hardening strain respectively.

### 2.6. Material Model of the Steel External Confinements

Figure 4b shows the bi-linear steel stress–strain model with linear strain hardening that was used in this study to establish the stress–strain curves of the ring and spiral external confinements. The strain hardening modulus was calculated as suggested by Pagoulatou et al. [32] (*E_sh_* = *E_s_*/100). The external confinements ultimate strength (*f_ue_*) was calculated according to the following Equation (3) suggested by Tao et al. [31].

(3) $$f_{ue}=\left[1.6-9.17\times 10^{-4}\left(f_{ye}-200\right)\right]f_{ye}\;\;\;\;\;\;\;\;200\leq f_{ye}\leq 800\;\mathrm{Mpa}$$

Poisson’s ratio (*ν_p_* = 0.3) and elastic modulus of external confinements (*E_s_* = 200 GPa) were taken as suggested by Tao et al. [31].

### 2.7. Material Model of the Concrete Core

The ABAQUS ‘concrete damaged plasticity model’ (CDP) was adopted to describe the material performance of the concrete infill. This model provides the ability to simulate the behaviour of quasi-brittle materials including concrete. To utilize this model, a few main parameters should to be defined including the concrete dilation angle (*ψ*), the compressive meridian (*K_c_*), potential eccentricity (*e*) and the relationship of the compressive strength under biaxial loading to uniaxial compressive strength (*f_bo_*/*f_c_^′^*). The parameters were determined as recommended by Tao [27], where the values of ψ and Kc were calculated using Equations (4) and (5), respectively. In addition, the default value of 0.1 was taken to define the flow potential eccentricity (*e*).

(4) $$\psi =\{\begin{array}{ll} 56.3\left(1-\xi_{c}\right) & \mathrm{for}\;\xi_{c}\leq 0.5\\ 6.672e^{\frac{7.4}{4.64+\xi_{c}}} & \mathrm{for}\;\xi_{c}>0.5 \end{array}$$

(5) $$K_{c}=\frac{5.5}{5+2{\left(f_{c}^{\prime }\right)}^{0.075}}$$

The ratio of *f_bo_*/*f_c_^′^* was calculated using Equation (6) as suggested by Papanikolaou and Kappos [33].

(6) $$f_{b0}/f_{c}^{\prime }=1.5{\left(f_{c}^{\prime }\right)}^{-0.075}$$

**Figure 5:**
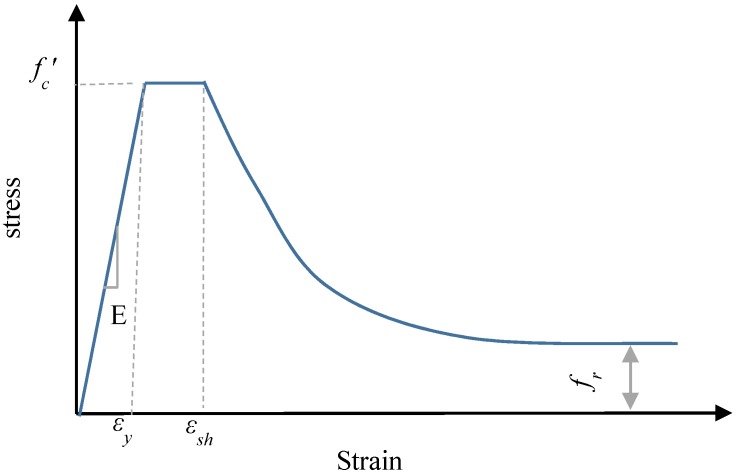
Concrete stress–strain model.

For the elastic part of the stress–strain relationship of the concrete core, the concrete modulus of elasticity was calculated as recommended by ACI 318 [34] (*E_c_* = 4700$\sqrt{f_{c}^{\prime }}$). The Poisson’s ratio for concrete was 0.2 as it has been widely used in previous simulation studies [35,36]. The stress–strain relationship model illustrated in Figure 5 as suggested by Tao et al. [27] was used to simulate the compressive and tensile properties of the confined concrete. The model was established based on an extensive range of experimental tests of CFST members.

### 2.8. Model Validation

The accuracy of the generated FE model was verified by comparing the FE results against the test results (88 specimens) that been conducted previously by other researchers [21,22,30]. Table 2 and Table 3 show a comparison of the ultimate strength obtained from the experimental testing results (*N_Exp_*) and FEA simulation results (*N_FEA_*). A reasonable agreement was gained between the predicted and test outcomes. In this paper, the CFST ultimate strength was determined as the first peak load, while when the specimens exhibited a strain hardening performance, the ultimate strength was calculated as the strength corresponding to 5% axial strain for the specimens [21,22,30].

**Figure 6:**
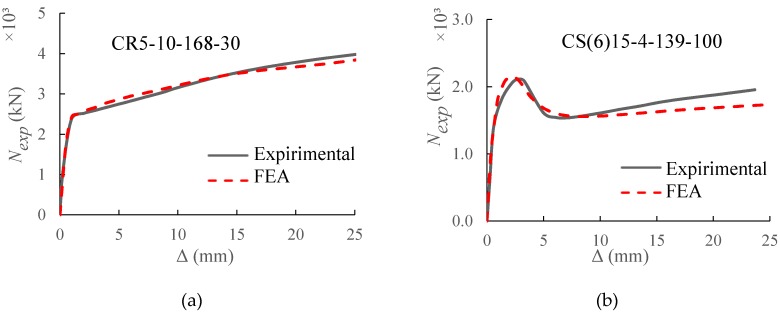
Axial load (*N_exp_*) versus axial deformation (Δ) curves of the CFST specimens with external (**a**) ring and (**b**) spiral confinements.

For CFST specimens with ring and spiral confinements, the mean values of *N_Exp_*/*N_FEA_* are 1.028 and 1.002 with corresponding standard error (SE) of 0.005 and 0.012, respectively. Figure 6a,b present the comparison between the measured and FE axial load-displacement relationships for the CFSTs with external rings (CR5-10-168-30 [21]) and spiral (CS(6)15-4-139-100 [22]) confinement, respectively. Similarly, and for the a 90 CFST specimens, a good agreement was observed of the load-displacement relationship of the suggested FE model the experimental results.

**Figure 7:**
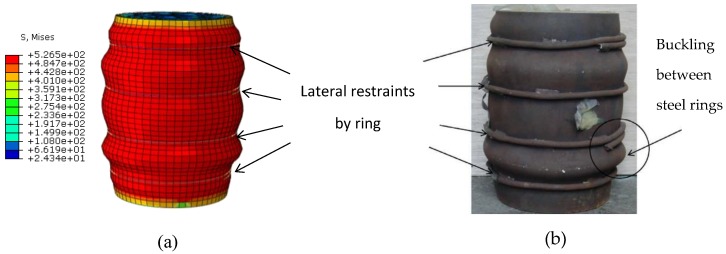
Comparison of observed and predicted failure mechanism. (**a**) Predicted; (**b**) Test (CR10-8-168-30) [21].

In order to have more confidence in the reliability of the proposed FE model, Figure 7 illustrates a comparison between the experimental and FEA failure mechanism for column specimen CR10-8-168-30 [21]. An agreement was achieved between the experimental and predicted deformed shapes.

## 3. Analytical Behaviour

Three typical CFST stub columns with external rings, external spiral, and without external confinements were modelled in order to investigate their analytical behaviour. The columns parameters are as follows: specimen height (*L*) = 330 mm, specimen outer diameter (*D*) = 150 mm, tube thickness (*t*) = 4 mm, unconfined concrete cylinder strength (*f_c_^′^*) = 40 MPa, tube yield strength (*f_y_*) = 400 MPa, external confinement yield strength (*f_ye_*) = 350 MPa, and external confinement diameter (*d*) = 8 mm.

### 3.1. Typical Failure Mode

**Figure 8:**
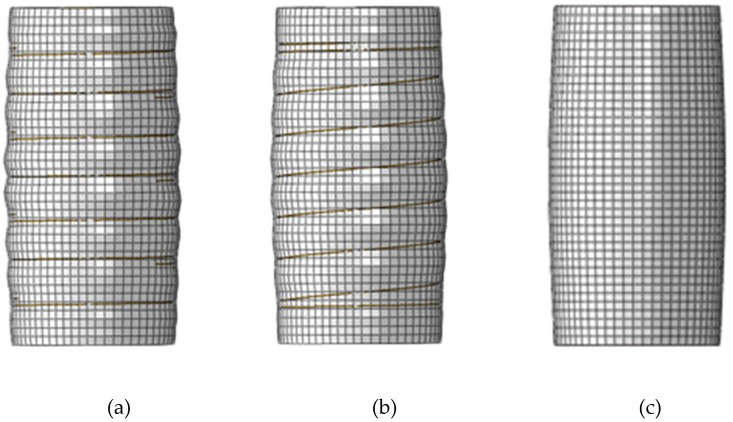
Typical failure mode of the CFSTs with and without external confinements. (**a**) Column with external rings; (**b**) Column with external spiral; (**c**) Column without external confinement.

Figure 8 shows the typical failure mechanism for CFSTs with and without external confinements under axial loading. An outward buckling mode is observed for all cases. For the column without external confinements, the outward bulge is noted in the middle column. For the columns with outer rings and spiral, the buckling occurs between the external confinements. In addition, it was noted that the external steel confinement can offer a significant lateral restraint and minimise the effective depth of steel tube within the spacing between external stiffeners.

### 3.2. Load-Deformation Curves

**Figure 9:**
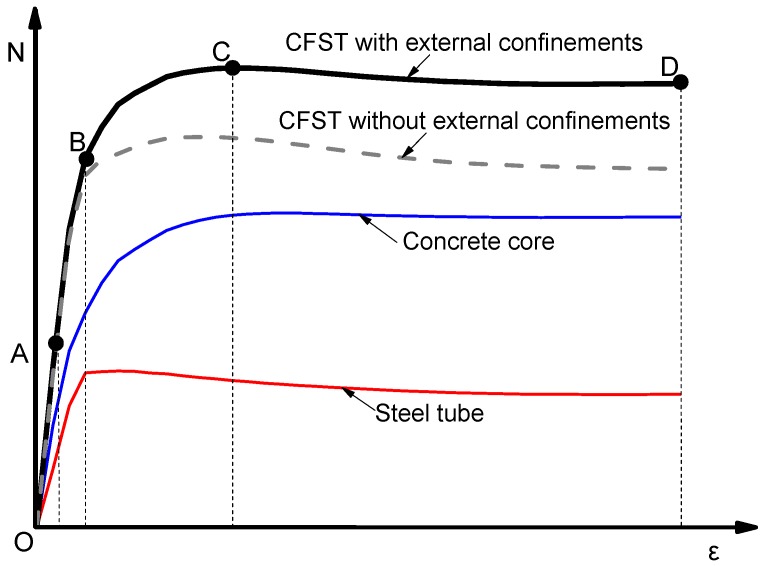
Typical *N-ε* relationship.

**Figure 10:**
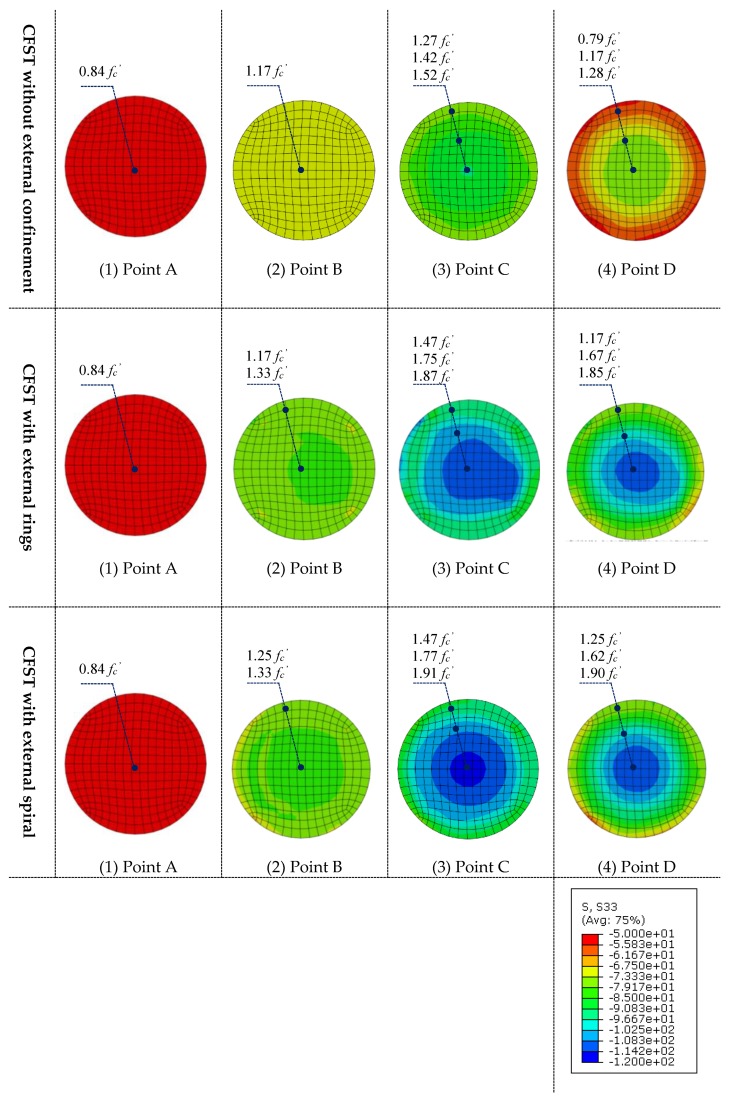
Longitudinal stress distribution of concrete infill.

The typical relationship between axial load (*N*) and axial strain (*ε*) of CFST columns with external confinements was calculated and presented in Figure 9. In addition, the axial load (*N*) carried by the column components including steel tube and the concrete infill, were also illustrated versus the corresponding strain (*ε*). In Figure 9, the curve is marked by four characteristic points to identify the different loading stages of the composite column. In addition, the longitudinal stress distribution (S33) of the concrete core at the mid-height of specimens was captured at the characteristic points and presented in Figure 10.

The behaviour of these columns is summarised into four main stages as the following:

**Stage 1** (from point O to A, Figure 9): In this stage, the column and its components show a linear elastic behaviour. For columns with and without external confinement, the steel tube and concrete carry the axial load independently. At point A (Figure 10), a consistently uniform distribution of concrete longitudinal stress was observed across all cross-sections for all cases. At point A, the longitudinal stresses of concrete core and steel tube are around 0.84*f_c_*′ and 0.74*f_y_* respectively for all columns cases.

**Figure 11:**
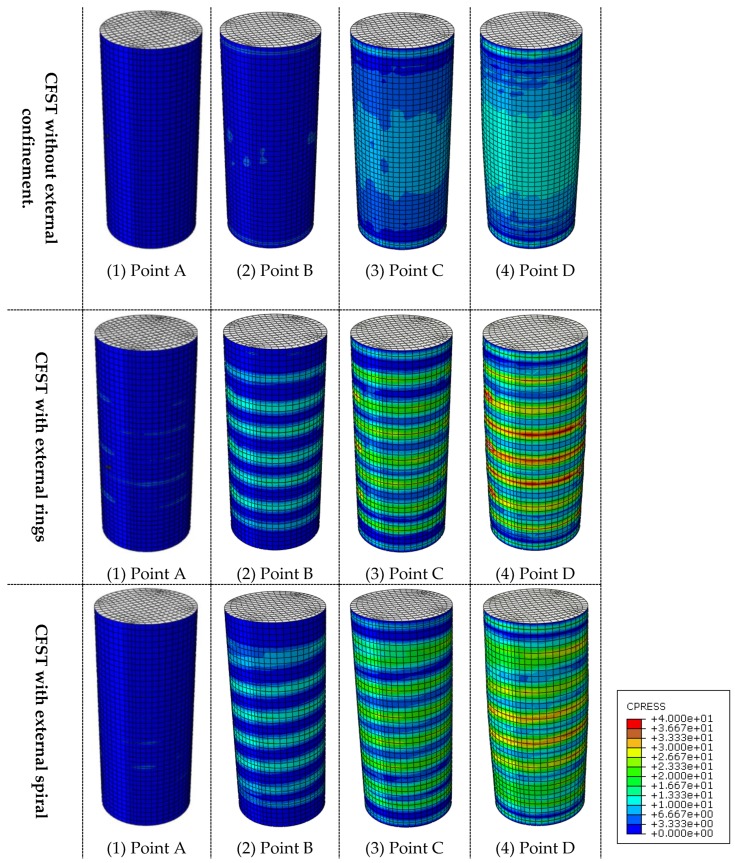
Stress distributions of steel-concrete interaction.

**Stage 2** (from point A to B, Figure 9): After point A, the composite column enters the elastic-plastic stage. During this stage, the slope of *N-ε* curve becomes less steep because of the concrete cracks that begin at point A. At point B, the tube reaches its yield strength which is before the ultimate strength point of the concrete core and the whole composite column. As illustrated in Figure 11 at this point (B), the interaction between the concrete and steel becomes considerable for the columns with external confinement because of the lateral expansion of concrete at this stage. However, the composite interaction is neglectable for columns without external confinement due to the relatively uncontrolled lateral dilation of the unconfined steel tube.

**Stage 3** (from point B to C, Figure 9): The columns show plastic behaviour in this stage. In addition, the contact pressure between the steel and concrete is significantly growing after point B until point C, where the columns reached their ultimate axial strength. For all cases at point C in Figure 10, the concrete longitudinal stress increases as it is closer to the centre of the concrete core. This is due to the confinement provided by the steel tube. However, the confinement strength is higher for columns with external confinement. In addition, a relatively uneven distribution of concrete longitudinal stress was observed for CFST with external rings, this is due to the non-symmetry resulting from the existing overlap length of the steel rings.

**Stage 4** (from point C to D, Figure 9): When the axial strain increases, the axial load resisting began to decline until it reaches point D where the FEA calculation is terminated because of the relatively stable load.

### 3.3. Interaction Behaviour

**Figure 12:**
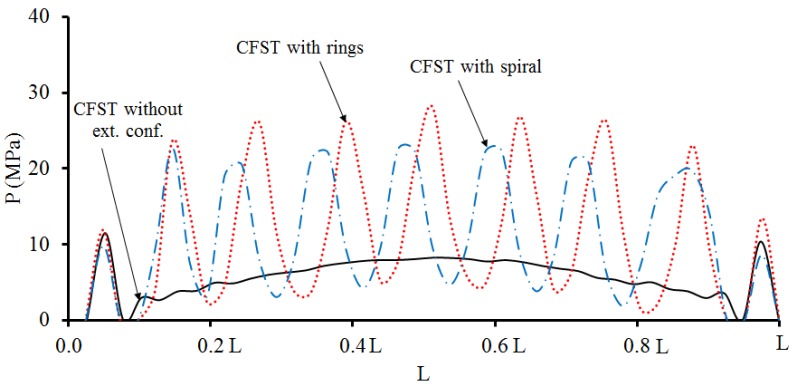
Steel-concrete interaction stress (*P*) along the CFST column at point C.

The bond or interaction stresses (*P*) is the stress acting on the interface of concrete core to the inner surface of the steel tube. Figure 12 shows the distribution of *P* along the CFST column. The *P* values were numerically calculated at point C when the columns reach their ultimate strength. The presence of external confinements has a positive impact in enhancing the steel–concrete composite behaviour, which is directly related to the concrete confinement effects and can improve the overall performance of the CFST columns.

**Figure 13:**
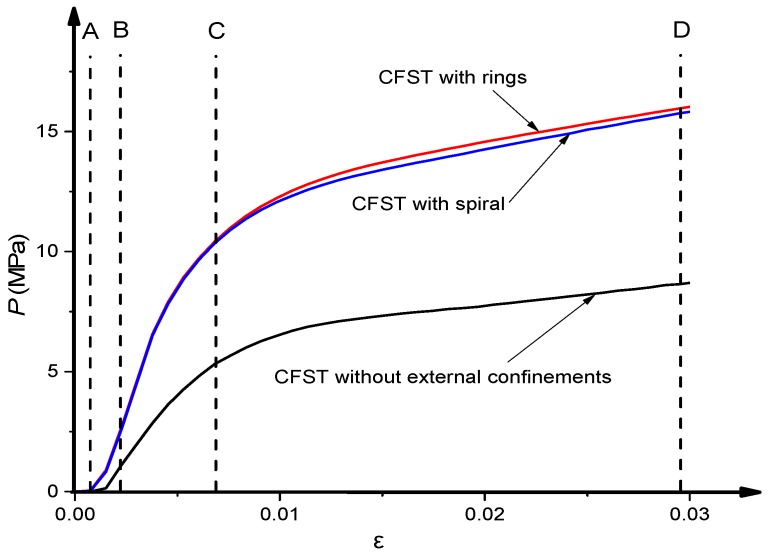
Impact of external confinements on *P-ε* relationship.

Figure 13 shows the influence of adopting external steel confinements on the *P-ε* relationship. The interaction stresses (*P*) are the average values of interaction stresses generated on the steel-concrete contact surface. For all CFST columns, there is no interaction developed at the elastic loading stage of the composite columns (from O to A). At this stage, the steel tubes have larger lateral expansion than concrete infill due to the difference in Poisson’s ratios for the two materials. The steel-concrete interaction begins to emerge after point A when the concrete begins cracking and bulge outward. The contact stress (*P*) value has significantly increased during the following loading stages, especially with the presence of external confinements.

**Figure 14:**
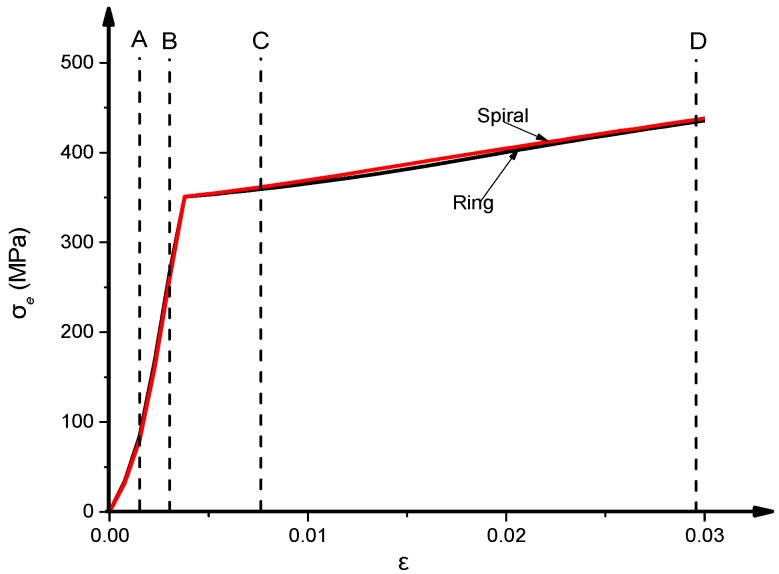
External confinement stress (*σ_e_*)–*ε* relationship.

Figure 14 shows the relationship of the external confinements stress (*σ_e_*) versus axial strain (*ε*) in the middle-height section of the CFST column. Overall, the CFST columns with external rings or spiral confinements have almost identical *σ_e_*-*ε* relationship. The *σ_e_* value develops from the initial loading stage at point O and still exists during the entire loading stages. The external confinements begin to yield after point B when the composite column reaches the plastic stage.

## 4. Parametric Analysis

Based on the verified FEA, extensive parametric analysis was carried out to have a better understanding of the performance of CFST stub columns with external confinements. The analysis investigated the yield strength (*f_ye_*), horizontal spacing (*S*), and diameter (*d*) of external confinements were the main parameters of this analysis. In addition, other parameters were considered including concrete strength (*f_c_^′^*), steel tube yield strength (*f_y_*), and diameter to thickness ratio (*D*/*t*). In each simulation case, the basic parameters were taken as suggested in the analytical behaviour investigations (Section 3) except the parameter under consideration.

### 4.1. Influence of Concrete Grade

**Figure 15:**
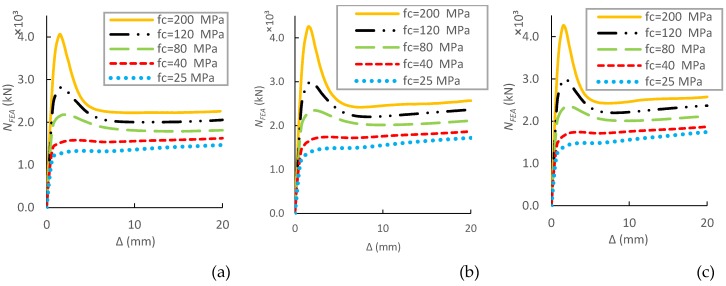
Parametric analysis on the influence of *f_c_^′^* on *N–∆* relations of CFSTs. (**a**) without external confinements; (**b**) With ring confinements; (**c**)With spiral confinements.

This section investigated the impact of concrete compression strength (*f_c_^′^*) on the fundamental performance and ultimate strength of CFST short columns with external confinements by increasing the concrete grades from 20 to 200 MPa. As shown in Figure 15, the increase of *f_c_^′^* results in a linear growth of load-carrying capacity, whereas the post-peak performance shows a decrease in ductility.

**Figure 16:**
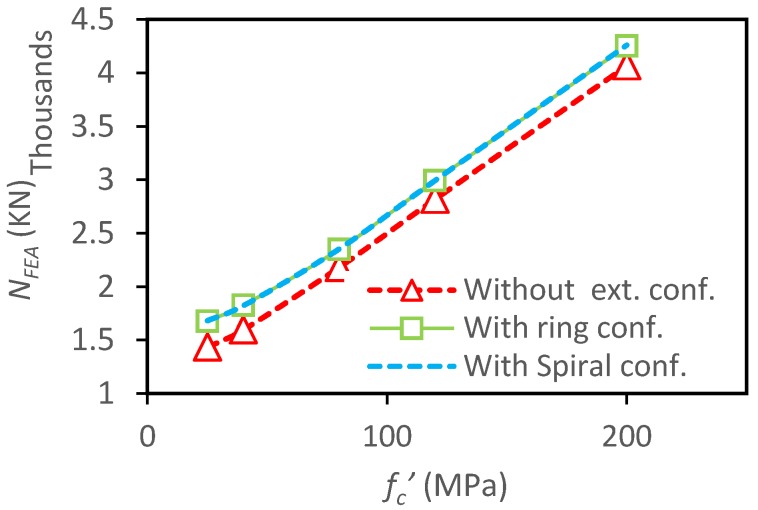
Parametric analysis on the influence of *f_c_^′^* on load ultimate capacity of CFSTs.

The specimens with external rings and spiral produce similar increases in the load-carrying capacity with the increase of *f_c_^′^* (Figure 16) when compared to CFSTs without external confinements. The corresponding increase rates of ultimate capacity are 17%, 14%, 8%, 6%, and 4.5% for both ring and spiral columns with concrete strength of 25 MPa, 40 MPa, 80 MPa, 120 MPa, and 200 MPa, respectively. Obviously, the external confinements have lower influence on columns with higher concrete strength because higher strength concrete has lower lateral expansion at the early loading phase [37,38].

### 4.2. Influence of Steel Tube Grade

**Figure 17:**
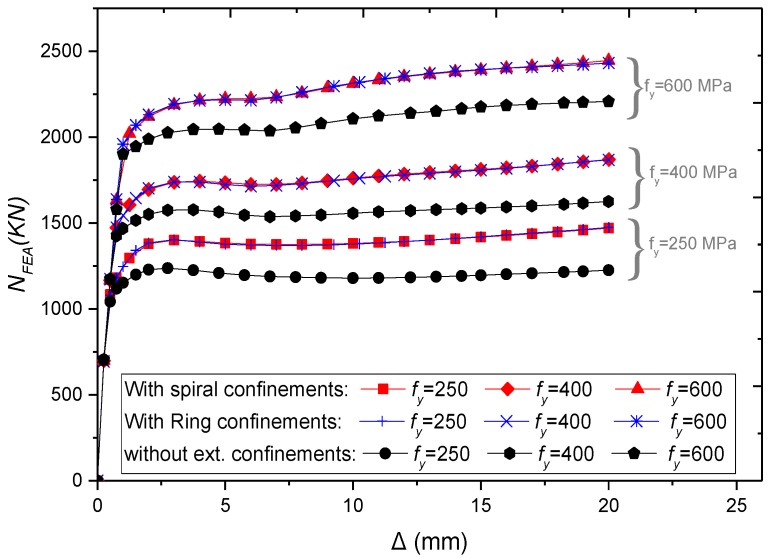
Parametric analysis on the influence of *f_y_* on *N_FEA_–∆* relations of CFSTs.

**Figure 18:**
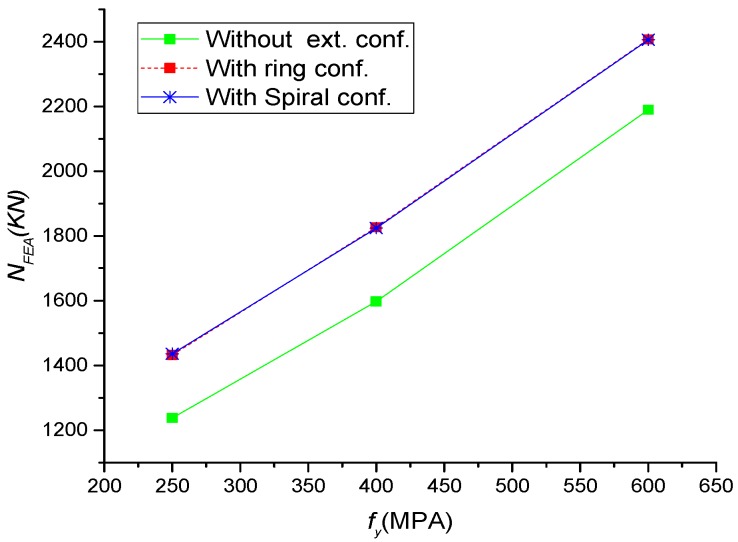
Parametric analysis on the effect of *f_y_* on load ultimate strength of CFSTs.

The yield strength of the steel tube (*f_y_*) was taken as 250MPa, 400MPa, and 600 MPa. As expected, Figure 17, the column ultimate strength increases linearly with the increase of *f_y_*, while there was a slight improvement in ductility. Additionally, an identical performance was observed for specimens with external rings and spiral (Figure 18). The external confinements have increased the ultimate strength by 16%, 14%, and 10% for columns with the steel strength of 250 MPa, 400 MPa, and 600 MPa, respectively.

### 4.3. Influence of Diameter-to-Thickness (D/t) Ratio

In order to study the impact of *D*/*t* ratio on the general behaviour of CFSTs with external confinements, the thickness of the tube was changed to get the *D*/*t* ratio ranging from 15 to 150 while the diameter of the steel tube is unchanged.

**Figure 19:**
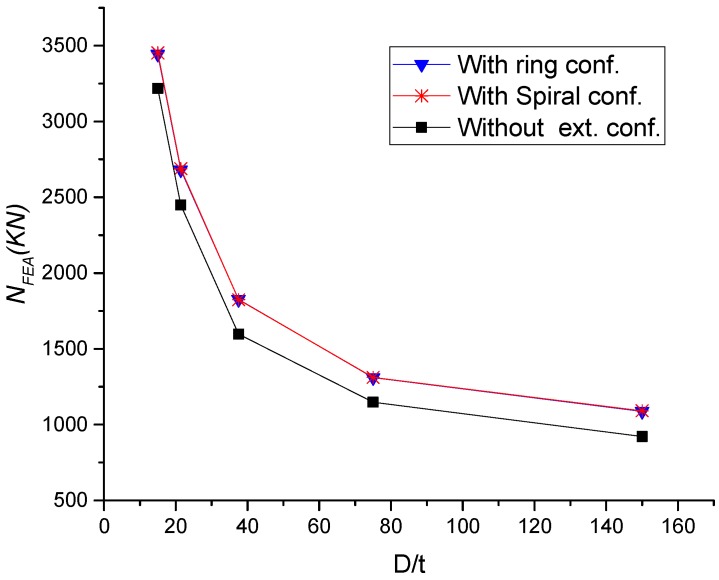
Parametric analysis on the influence of *D*/*t* ratio on load ultimate capacity of CFST columns.

**Figure 20:**
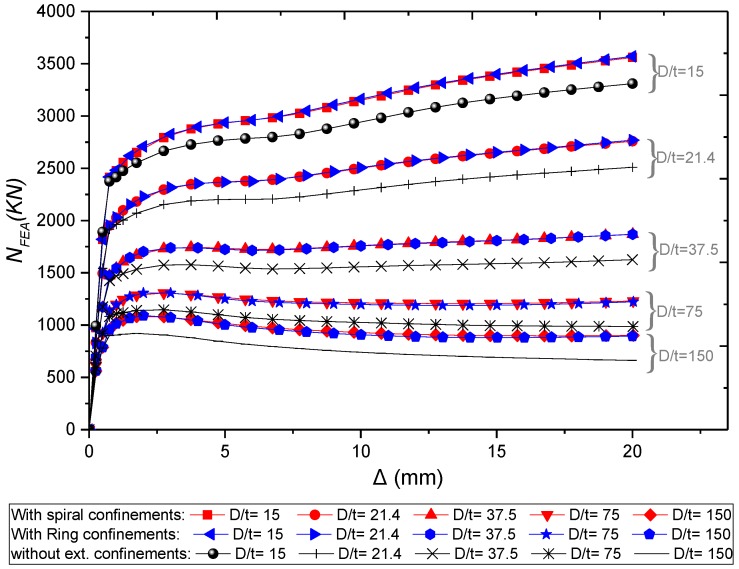
Parametric analysis on the influence of *D*/*t* ratio on *N_FEA_ –∆* relations of CFSTs.

Similar to the previously mentioned parameters, the ring and spiral columns have almost identical performance. Figure 19 and Figure 20 show that any decrease in *D*/*t* ratio can result in significant improvement in the load-carrying capacity and ductility. This consequence was expected where any reduction of steel thickness will affect the concrete confinement by the tube. For specimens with external confinements, the increase of *D*/*t* ratio from 15, 21.4, 37.5, 75 and 150, lead to increase the load-carrying capacity by about 7%, 10%, 14 %, 14%, and 18%, respectively, compared to columns without external confinements.

### 4.4. Influence of External Confinements Grade

**Figure 21:**
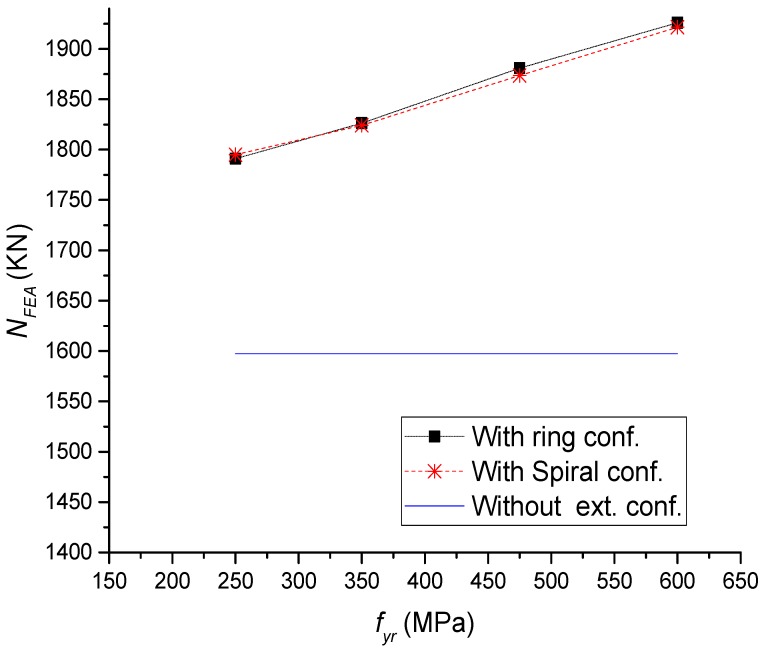
Parametric analysis on the influence of *f_ye_* on load ultimate capacity of CFST columns.

**Figure 22:**
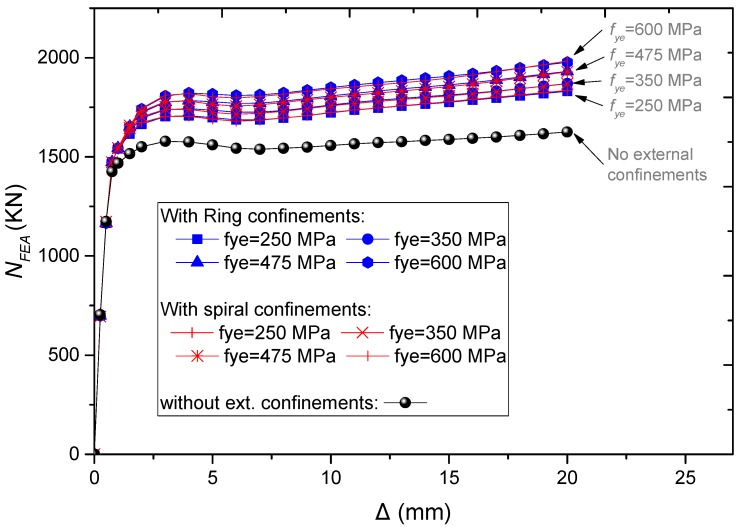
Parametric analysis on the influence of *f_ye_* on *N–∆* relations of CFST columns.

The performance of CFST short columns with external confinements was examined for different strengths of external confinements. For both rings and spiral external confinements, the increase of *f_ye_* to 250, 350, 475, and 600 has led to the increase of ultimate strength by 12%, 14%, 17%, and 20%, respectively, compared to columns without external confinements (Figure 21 and Figure 22).

### 4.5. Influence of Steel Ratio (α)

The previous parametric investigations adopted an equal spacing (*S* = 20 mm) between both external rings and spiral confinements. However, the amount of steel used in the outer confinements and the total steel ratio was different for columns with external ring and external spiral confinements. Therefore, the effect of the total steel ratio (*α*) on the load-carrying capacity and ductility was examined by changing the distances between the external confinements. In order to assess the efficiency of utilising external confinements rather than increasing the steel wall thickness of the CFST columns, the results were compared with CFST columns without external confinements that have the same steel ratio of 3.3%, 5.5%, and 8%.

**Figure 23:**
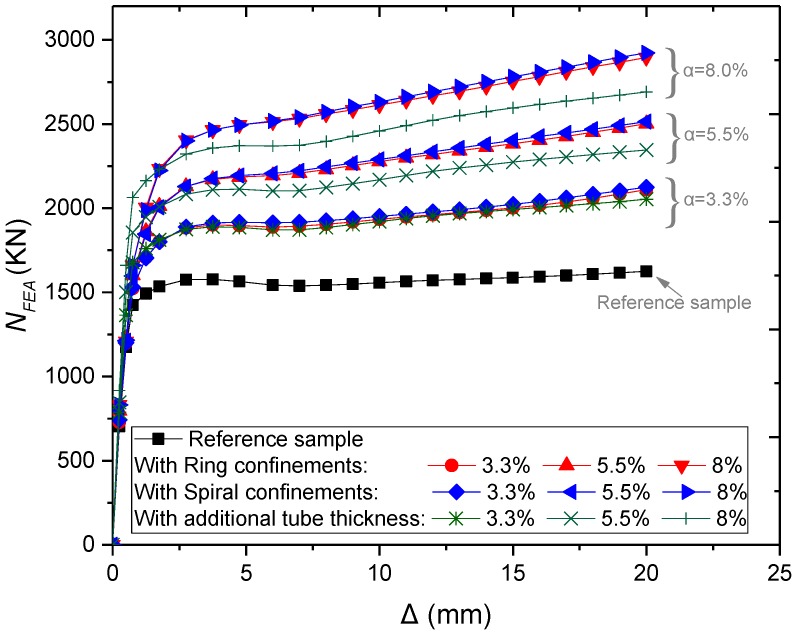
Parametric analysis on the influence of *α* on *N_FEA_ –∆* relations of CFSTs.

**Figure 24:**
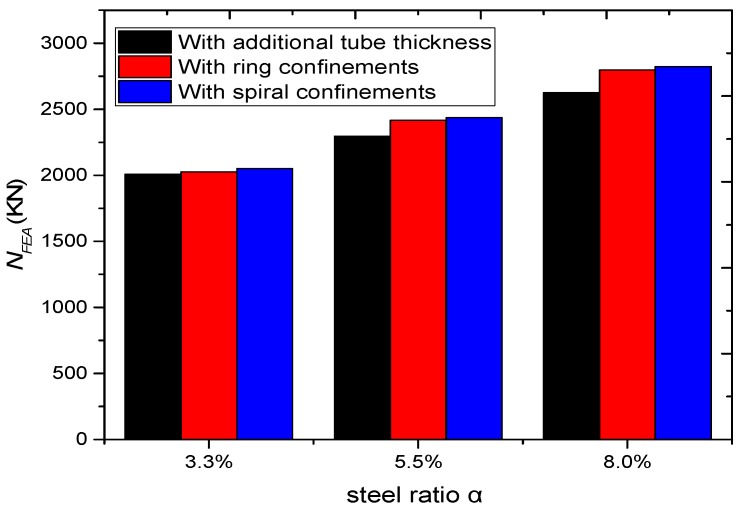
Parametric analysis on the influence of α on load ultimate capacity of CFSTs.

The simulation results (Figure 23) show that *α* increases the load-carrying capacity and ductility of the columns increases. The structural behaviour of CFSTs with spiral external confinements is better because spirals can act as one element and provide relatively uniform confinement. However, this performance remained relatively close to the performance of the ring confined columns. Besides that, the performance is better than columns without external confinements that have the same ratio of steel (Figure 24).

## 5. Prediction of the Ultimate Strength

To simplify the design calculation, the external confinements are transformed into circular steel tube using equivalent wall thickness (*t_e_*) that have the same steel ratio.

(7) $$t_{e}=\sqrt{\left(A_{st}+A_{c}\right)/\pi }-D/2$$

where *A_st_* is the total equivalent steel area and can be calculated using Equations (8) and (9) for columns with external ring and spiral confinements, respectively.

(8) $$A_{st}=A_{s}+\frac{n\;d^{2}}{4H}\left(D+d\right)\frac{f_{yr}}{f_{y}}$$

(9) $$A_{st}=A_{s}+\frac{\pi d^{2}}{4H}\left(2\pi \left(D+d\right)+n\;\sqrt{S^{2}+\pi^{2}{\left(D+d\right)}^{2}}\right)\frac{f_{yr}}{f_{y}}$$

**Figure 25:**
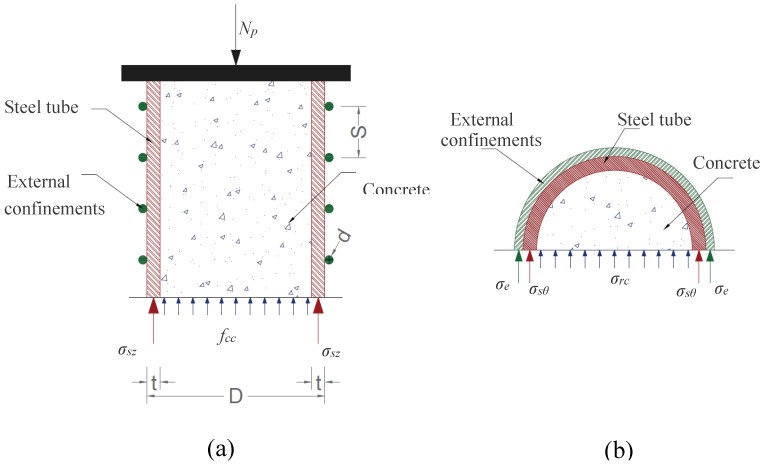
Stress state of CFST columns: (**a**) axial direction; (**b**) free body diagram.

As shown in Figure 25a, the axial load capacity of CFST columns (*N_p_*) can be obtained based on the static equilibrium of the section:

(10) $$N_{p}=\sigma_{sz}A_{st}+f_{cc}A_{c}$$

where *σ_sz_* is the axial stresses of the steel tube and was estimated as *σ_sz_* = *f_y_* based on the FEA results as illustrated in Figure 9. *f_cc_* is the confining stress of concrete was calculated as suggested by Mander et al. [39]. Where *k* is a confinement coefficient and can be taken as 4.1 as proposed by Richart et al. [40]. Additionally, *σ_rc_* is the lateral confining stresses of the concrete infill and any increase of *σ_rc_* is improving the concrete compressive strength and thereby the axial capacity of the composite columns.

The stress of the external confinements (*σ_e_*) was taken as *σ_e_* = *f_ye_*, where the column reached its ultimate strength at point C after the yield of the external confinement as shown in Figure 14.

*σ_rc_* can be estimated based on the force equilibrium condition illustrated in Figure 25b.

(11) $$f_{cc}=f_{c}^{\prime }+k{\;\sigma }_{rc}$$

(12) $$\sigma_{rc}=\frac{2\sigma_{s\theta }{}_{\;}tH+f_{yr}\frac{\pi }{2}\;d^{2}n}{\left(D-2t\right)H}$$

Based on the actual and the parametric analysis results, the hoop stress of steel tube (*σ_sθ_*) can be calculated by Equation (13).

(13) $$\sigma_{s\theta }/f_{y}=\{\begin{array}{cc} 0.167\;\xi^{\prime } & 0<\xi^{\prime }\leq 1.95\\ 0.326 & 1.95<\xi^{\prime }\leq 4.90 \end{array}$$

The equivalent confinement index (*ξ′*) is calculated using Equation (14). This new proposed formula is adopted to take the influence of external confinement into account besides the impact of concrete infill and steel tube.

(14) $$\xi^{\prime }=\frac{A_{st}f_{y}}{A_{c}f_{c}^{\prime }}$$

**Figure 26:**
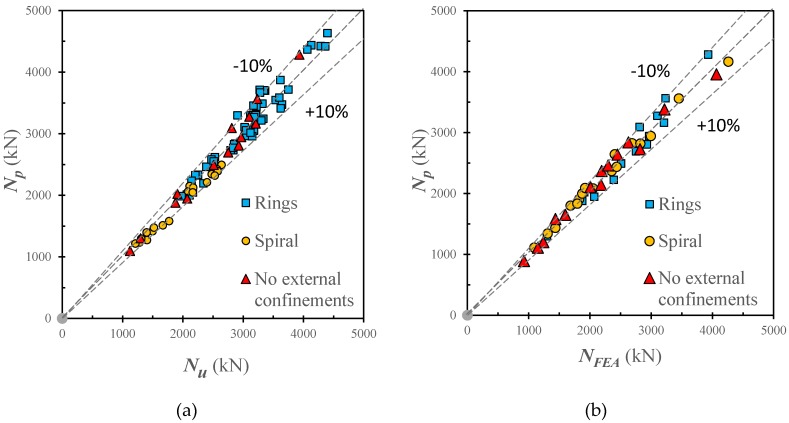
Comparison of load-carrying capacities obtained from the proposed calculation method, test results, and FEA results. (**a**) Measured results; (**b**) Numerical results.

Figure 26 compare the predicted ultimate capacities (*N_p_*) using Equation (10) against the experimental (*N_Exp_*) and FEA (*N_FEA_*) results. For the experimental data, the mean value (*μ*) of *N_Exp_*/*N_p_* is 1.00 with corresponding standard deviation (SD) standard error (SE) of 0.054 and 0.107 respectively. For the numerical data, the mean value of *N_e_*/*N_FEA_* is 0.978 with corresponding standard deviation (SD) standard error (SE) of 0.041 and 0.140 respectively. Therefore, the proposed calculation method can accurately predict the ultimate strength of CFSTs with and without external steel confinements.

## 6. Conclusions

The following are the conclusions based on the numerical investigations on the axial performance of CFST stub columns with and without external steel confinements:

- A finite element (FE) model was established to examine the behaviour of circular CFST stub columns with and without external steel confinement which is subjected to axial loading. The accuracy of FE results was validated based on previous experimental tests. Good agreement was achieved between the numerical and test results.
- This study described the analytical behaviour of CFSTs with and without external steel confinements, and the axial load (*N*) and axial strain (*ε*) relationship was divided into four main stages. During the elastic stage, *N-ε* curves were almost the same for CFSTs with and without external confinements. After that, CFSTs with external confinement show higher strength and better ductility.
- The presence of external confinements can remarkably improve the steel-concrete interaction stress, especially after the elastic stage.
- According to the results parametric analysis, the increase of concrete strength, steel tube yielding strength, external confinements yielding strength, and total steel ratio besides the decrease of diameter-to-thickness ratio lead to enhance the structural performance of CFST columns at varying rates.
- Under axial loading, the use of external steel confinements in CFST can provide better performance than increasing the thickness of steel tube when using the same steel ratio.
- A simplified design method was developed to accurately estimate the ultimate strength of CFST columns with and without external steel confinement.
- Future investigation needs to consider the influence of different slenderness ratio, cross-sectional shapes, and materials of CFST columns with external steel confinements under different loading conditions.

## Figures and Tables

**Table 1:** Effect of load rating and mass scaling on CPU time (for CPU @ 2.00 GHz Core i7 with 8 GB RAM).

Loading Rate	Mass Scaling Factor	Increment	CPU Time (s)
6500 mm/s	10	5048	134
2500 mm/s	10	12558	306
500 mm/s	10	62190	2152
500 mm/s	5	87758	2521
500 mm/s	30	35985	1279

**Table 2:** Test data of CFST stub columns with ring external confinements [21,30].

Group No.	Specimens	*N_exp_* (kN)	*N_FEA_* (kN)	$\frac{N_{exp}}{N_{FEA}}$
R1	CR5-5-168-30	2836	2736	1.037
	CR10-5-168-30	2387	2464	0.969
	CR12.5-5-168-30	2250	2335	0.964
	CR15-5-168-30	2205	2327	0.948
	CR20-5-168-30	2142	2239	0.957
	CN0-5-168-30	1908	2024	0.943
R2	CR5-8-168-30	3536	3550	0.996
	CR10-8-168-30	3217	3358	0.958
	CR12.5-8-168-30	3163	3309	0.956
	CR15-8-168-30	3117	3300	0.945
	CR20-8-168-30	2905	3300	0.880
	CN0-8-168-30	2810	3093	0.908
R3	CR5-10-139-30	2791	2733	1.021
	CR10-10-139-30	2530	2621	0.965
	CR15-10-139-30	2473	2584	0.957
	CR20-10-139-30	2511	2550	0.985
	CN0-10-139-30	2510	2495	1.006
R4	CR5-10-168-30	3616	3874	0.934
	CR10-10-168-30	3364	3702	0.909
	CR12.5-10-168-30	3346	3707	0.903
	CR15-10-168-30	3273	3715	0.881
	CR20-10-168-30	3278	3671	0.893
	CN0-10-168-30	3232	3566	0.906
R5	CR5-10-139-50	3038	2966	1.024
	CR10-10-139-50	2866	2842	1.009
	CR15-10-139-50	2849	2831	1.006
	CR20-10-139-50	2835	2772	1.023
	CN0-10-139-50	2750	2695	1.020
R6	CR5-5-168-80	3643	3475	1.048
	CR10-5-168-80	3205	3166	1.012
	CR12.5-5-168-80	3178	3052	1.041
	CR15-5-168-80	3079	3067	1.004
	CR20-5-168-80	3149	2960	1.064
	CN0-5-168-80	2926	2809	1.042
R7	CR5-8-168-80	3749	3720	1.008
	CR10-8-168-80	3317	3489	0.951
	CR12.5-8-168-80	3600	3589	1.003
	CR15-8-168-80	3218	3443	0.935
	CR20-8-168-80	3171	3459	0.917
	CN0-8-168-80	3101	3278	0.946
R8	CR5-10-139-90	3333	3245	1.027
	CR10-10-139-90	3022	3108	0.972
	CR15-10-139-90	3047	3056	0.997
	CR20-10-139-90	3120	3018	1.034
	CN0-10-139-90	2966	2944	1.007
R9	CR5-10-168-90	4396	4635	0.948
	CR10-10-168-90	4130	4439	0.930
	CR12.5-10-168-90	4285	4426	0.968
	CR15-10-168-90	4361	4420	0.987
	CR20-10-168-90	4063	4370	0.930
	CN0-10-168-90	3930	4284	0.917
R10	CR5-5-114-120	2340	2195	1.066
	CR10-5-114-120	2167	2047	1.059
	CR12.5-5-114-120	2065	2016	1.024
	CR15-5-114-120	2110	2002	1.054
	CR20-5-114-120	1977	1987	0.995
	CN0-5-114-120	1875	1881	0.997
R11	CR5-10-139-120	3621	3413	1.061
	CR10-10-139-120	3207	3305	0.970
	CR15-10-139-120	3180	3272	0.972
	CR20-10-139-120	3301	3218	1.026
	CN0-10-139-120	3208	3164	1.014

**Table 3:** Test data of CFST stub columns with spiral external confinements [22,30].

Group No.	Specimens	*N_exp_* (kN)	*N_FEA_* (kN)	$\frac{N_{exp}}{N_{FEA}}$
S1	CS(6)10-4-139-30	1403	1274	1.112
	CS(6)15-4-139-30	1278	1235	1.046
	CS(8)20-4-139-30	1307	1298	1.017
	CS(6)20-4-139-30	1211	1219	1.004
	CN0-4-139-30	1122	1098	1.022
S2	CS(8)10-4-139-50	1770	1583	1.118
	CS(6)10-4-139-50	1512	1459	1.036
	CS(8)15-4-139-50	1665	1516	1.099
	CS(6)15-4-139-50	1496	1420	1.054
	CS(8)20-4-139-50	1518	1478	1.027
	CS(6)20-4-139-50	1396	1583	1.118
	CN0-4-139-50	1297	1302	0.996
S3	CS(8)10-4-139-100	2398	1.083	1.085
	CS(6)10-4-139-100	2128	1.008	1.010
	CS(8)15-4-139-100	2109	0.980	0.982
	CS(6)15-4-139-100	2086	1.009	1.011
	CS(8)20-4-139-100	2171	1.021	1.023
	CS(6)20-4-139-100	2161	1.054	1.056
	CN0-4-139-100	2070	1949	1.062
S4	CS(8)10-4-139-120	2640	1.057	1.059
	CS(6)10-4-139-120	2488	1.047	1.048
	CS(8)15-4-139-120	2566	1.058	1.060
	CS(6)15-4-139-120	2476	1.056	1.057
	CS(8)20-4-139-120	2577	1.077	1.078
	CS(6)20-4-139-120	2528	1.089	1.090
	CN0-4-139-120	2390	2226	1.074
